# Ginsenoside Rg3 inhibits pulmonary fibrosis by preventing HIF-1α nuclear localisation

**DOI:** 10.1186/s12890-021-01426-5

**Published:** 2021-02-27

**Authors:** Zhuo Fu, Yong-sheng Xu, Chun-quan Cai

**Affiliations:** 1 Tianjin Medical University, Tianjin, China; 2 Department of Respiratory, Tianjin Children’s Hospital, Tianjin, China; 3 Department of Neurosurgery, Tianjin Institute of Pediatrics, The Children’s Hospital of Tianjin, No.238 Longyan Road, Beichen District, Tianjin, 300400 China

**Keywords:** Ginsenoside, Pulmonary fibrosis, HIF-1α, Epithelial mesenchymal transition

## Abstract

**Background:**

Excessive fibroblast proliferation during pulmonary fibrosis leads to structural abnormalities in lung tissue and causes hypoxia and cell injury. However, the mechanisms and effective treatment are still limited.

**Methods:**

In vivo, we used bleomycin to induce pulmonary fibrosis in mice. IHC and Masson staining were used to evaluate the inhibitory effect of ginsenoside Rg3 in pulmonary fibrosis. In vitro, scanning electron microscopy, transwell and wound healing were used to evaluate the cell phenotype of LL 29 cells. In addition, biacore was used to detect the binding between ginsenoside Rg3 and HIF-1α.

**Results:**

Here, we found that bleomycin induces the activation of the HIF-1α/TGFβ1 signalling pathway and further enhances the migration and proliferation of fibroblasts through the epithelial mesenchymal transition (EMT). In addition, molecular docking and biacore results indicated that ginsenoside Rg3 can bind HIF-1α. Therefore, Ginsenoside Rg3 can slow down the progression of pulmonary fibrosis by inhibiting the nuclear localisation of HIF-1α.

**Conclusions:**

This finding suggests that early targeted treatment of hypoxia may have potential value in the treatment of pulmonary fibrosis.

**Supplementary Information:**

The online version contains supplementary material available at 10.1186/s12890-021-01426-5.

## Background

Idiopathic pulmonary fibrosis is a severe interstitial lung disease that can cause progressive loss of the lung function and has high lethality [1, 2]. Pulmonary fibrosis is an end-stage change in a large class of lung diseases characterised by abnormal fibroblast proliferation, extracellular matrix accumulation with inflammatory damage and tissue structure destruction. The alveolar tissue of patients is damaged and abnormally repaired, causing scarring [1, 3, 4]. During pulmonary fibrosis, the abnormal proliferation of fibroblasts is similar to the biological behaviour of cancer [5–7]. Pulmonary fibrosis severely affects the human respiratory function. The respiratory function of patients continues to deteriorate as the disease and lung injury worsen. Idiopathic pulmonary fibrosis has a higher mortality rate than most tumours and is called a ‘tumour-like disease’ [4, 8, 9].

Pulmonary fibrosis and tumours have similar biological characteristics, hypoxia and excessive cell proliferation. Rapid tumour growth causes hypoxia in the internal tissue cells of solid tumours. Hypoxia stimulates will endow tumour cells a strong ability to migrate and lead to distant metastasis [10, 11]. In addition, tumour cells obtain more nutrients by mimicking the structure of blood vessels, thus showing vascular mimicry [12, 13]. Similarly, in pulmonary fibrosis, the alveoli are replaced by fibrotic cells, which prevents oxygen exchange and causes widespread hypoxia [14]. However, the relationship between hypoxia and fibroblast hyperproliferation remains unclear.

Ginsenoside Rg3 is the main active component of ginseng and has a variety of pharmacological effects, including anti-oxidant, anti-inflammatory and anti-tumor activities [15–17]. Therefore, we speculate that ginsenoside Rg3 may alleviate pulmonary fibrosis. Here we found an abnormal expression of HIF-1α in bleomycin-induced pulmonary fibrosis. The nuclear localization of HIF-1α can enhance cell migration and proliferation through the epithelial–mesenchymal transition (EMT) pathway. In addition, ginsenoside Rg3 can bind and inhibit the nuclear localisation of HIF-1α. Through in vitro and in vivo experiments, we verified that ginsenoside Rg3 inhibits excessive fibroblast proliferation through the HIF-1α pathway. These observations suggest that HIF-1α may serve as a potential molecular target for the hypoxic treatment of pulmonary fibrosis.

## Methods

### Cell lines

The lung fibroblast cell line LL 29 was obtained from ATCC. The cells were cultured in F12K minimum essential medium containing 15% fetal bovine serum (Gibco, USA) in an environment containing 5% CO_2_ at 37 °C.

### Pulmonary fibrosis model and experimental design

BALB/c mice weighing 18–22 g was purchased from Charles River Laboratories. The mice were randomly divided into groups with eight mice each. Mice anesthetised with pentobarbital sodium (100 mg/kg) were administered with 5 mg/kg of bleomycin intra-tracheally. Control mice were treated with an equal volume of saline. A fibrosis model was successfully established after bleomycin treatment for 30 days and pathological examination of the lungs. 35 days after bleomycin treatment, we administered ginsenoside Rg3 intratracheally at a dose of 5 mg/kg. It is administered once every 3 days for a total of 5 times. After 28 days, the mice were euthanized through the intravenous injection of pentobarbital sodium at a final concentration of 100 mg/kg. The mice were checked, and death was confirmed by observing lack of respiration and cardiac output. Fresh lung tissue was harvested in surgical resection and weighed to calculate the lung coefficient (Lung wet weight/body weight × 100%). Then tissues were fixed or frozen for subsequent pathology testing. Euthanasia methods was in accordance to the proper practice of AVMA 2020. All procedures involving animals were approved and performed in accordance with the ethical standards of the Institutional Animal Care and Use Committee (IACUC) at Tianjin International Joint Academy of Biomedicine.

### Pathological analysis

After the embedded tissue was cut into 4 μm sections and deparaffinized, the sections were treated with 3% H_2_O_2_ for 10 min to block endogenous peroxidase activity. After antigen retrieval in a microwave oven, the sections were incubated with normal goat serum at 37 °C for 10 min. Afterwards, the sections were incubated with the following primary antibodies: SMA (Affinity, Changzhou, China), HIF-1α (Affinity, Changzhou, China), E-cadherin (Affinity, Changzhou, China) and Vimentin (Affinity, Changzhou, China). After 2 h, the sections were washed and incubated with a secondary antibody (Zhongshan Biotechnology Co., Ltd., Beijing, China) at 37 °C for 30 min. After staining with 3,3′-diaminobenzidine, the tissues sections were incubated with hematoxylin staining solution for 20 s, washed with tap water for 5 min, and observed for staining intensity under a microscope. Each experiment was performed in triplicate.

### Cell migration assay

The treated cells were seeded onto a 24-well plate and grown to 90% density at 37 °C with 5% CO_2_. A straight scratch was then made in the middle of the plate. After 24 h, cell migration and the migration rates were recorded with a microscope. Each experiment was performed in triplicate.

### Cell invasion assay

Matrigel is scattered in the upper chamber. The treated cells were seeded into the upper chamber and cultured in serum-free medium. Then, 10% FBS medium was added to the 24-well plate. After culturing in an incubator containing 5% CO_2_ at 37 °C for 16 h, the cells in the chamber were wiped. The passed cells outside the chamber are stained with 0.1% crystal violet and washed with running water. Then passed cells were photographed under a microscope and analysed for cell invasion ability. Each experiment was performed in triplicate.

### Colony formation assay

The treated cells were evenly seeded in 6-well plate at a final concentration of 1000 cells per well and then incubated for 2 weeks at 37 °C in an environment with 5% CO_2_. The medium was changed every three days. When a macroscopic clone appeared, the culture was terminated. Cells were washed with 1 × PBS and subsequently fixed and stained with GIEMSA. The number of clones in each group was determined. Each experiment was performed in triplicate.

### Western blot

The Nuclear and Cytoplasmic Protein Extraction Kit (Beyotime Biotechnology Co., Ltd. P0027) was used to extract nuclear and plasma proteins from the tissues and cells. All lysates contained protease inhibitors. The quantified proteins were separated by 10% SDS-PAGE. After transferring the protein to the PVDF membrane and blocking with 5% BSA, the PVDF membrane was blocked at room temperature with the following primary antibodies: HIF-1α (Affinity, Changzhou, China), TGFΒ1 (Affinity, Changzhou, China), SAMD3 (Affinity, Changzhou, China), p-SMAD3 (Affinity, Changzhou, China) E-cadherin (Affinity, Changzhou, China), Vimentin (Affinity, Changzhou, China), Lamin B (Affinity, Changzhou, China) and GAPDH (Affinity, Changzhou, China). GAPDH and Lamin B were used as loading controls. After 4 h, the excess primary antibody was removed, and the PVDF membrane was incubated with HRP-labelled secondary antibody at room temperature for 2 h. Protein intensity was detected with an Image Lab instrument (Bio-Rad, USA). Each experiment was performed in triplicate.

### Adeno-associated virus (AV) construction

The full-length HIF-1α cDNA fragment was synthesized (Genewiz, Beijing, China) and cloned into the pAOV-CMV-MCS-3FLAG AAV vector (OBiO, Shanghai, China). Co-transfected with helper plasmid into 293FT cells and packaged into adeno-associated virus overexpressing HIF-1α. These viruses are used to overexpress HIF-1α in animals.

### Biacore assay

Human HIF-1α cDNA was synthesized by Genwiz (Beijing, China) and cloned into PET-His prokaryotic protein expression plasmid using BamHI and NheI endonucleases. Biacore assay was performed with a Biacore 3000 instrument (GE Healthcare, Piscataway, NJ, USA). 50 mM NHS and 200 mM EDC were mixed in equal volumes and injected into a closed CMD500M chip (XanTec Bioanalytics) at a rate of 10 μl/min. The purified HIF-1α protein was diluted with sodium acetate buffer pH 5.0 and injected into the chip, and the remaining active groups were blocked with 1 M ethanolamine. Subsequently, RG3 was injected into the CM5 sensor chips at a rate of 30 μl/min. Data analysis was conducted using the BIA evaluation software. Each experiment was performed in triplicate.

### Molecular docking

The crystal structure of HIF-1α was downloaded from the PDB database (http://www.rcsb.org) and used to perform molecular docking with Rg3 by using the Sybyl X1.1 software.

### Scanning electron microscope inspection

After treatment, the cells were grown on climbing films. After 24 h, the cells were fixed and dehydrated in acetone/isoamyl acetate (1:1) and dried with a gradient concentration of acetonitrile. The cells were then coated with gold and photographed using a scanning electron microscope (LEO 1530 VP, Germany). Each experiment was performed in triplicate.

### Statistical analysis

Data were analysed with the SPSS 18.0 statistics software (SPSS Inc., Chicago, IL, USA) and presented as the mean ± standard deviations of the mean. Significant differences between two groups were compared using a Student’s *t* test. Comparisons among three or more groups were conducted using ANOVA with post hoc contrasts by Student–Newman–Keuls test. *p* < 0.05 was considered to indicate a statistically significant difference.

## Results

### Ginsenoside Rg3 inhibits bleomycin-induced pulmonary fibrosis

**Fig. 1:**
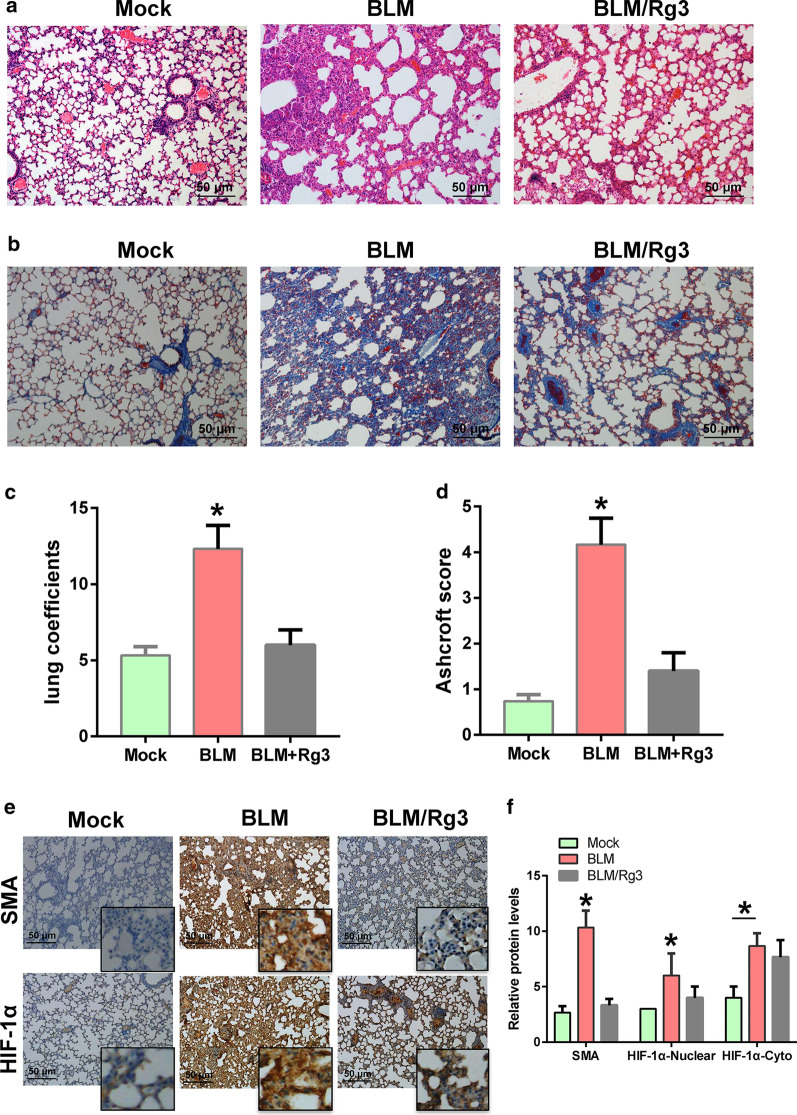
Rg3 inhibits bleomycin-induced pulmonary fibrosis. Potential therapeutic effects of Rg3 in mice with bleomycin-induced pulmonary fibrosis. **a** HE staining was used to detect the degree of pulmonary fibrosis (× 100 magnification). **b** Masson staining was performed to observe the fibroblasts (× 100 magnification). **c** Lung coefficients for evaluating pulmonary fibrosis. **d** Ashcroft score for evaluating pulmonary fibrosis. **e**, **f** Detection of SMA and HIF-1α expression by Immunohistochemical (× 100 magnification)

A total of 18 mice were randomly divided into three groups equally. The mice with bleomycin-induced pulmonary fibrosis were treated with ginsenoside Rg3 intratracheally at a dose of 5 mg/kg. After euthanasia, mouse lung tissue was obtained for pathological examination and fibrosis analysis. The HE staining results showed that the normal morphology of the lung tissue of the mice treated with bleomycin disappeared, while the alveolar structure of the mice was improved after Rg3 treatment (Fig. 1a). Masson staining results indicated that collagenous decreased after Rg3 treatment (Fig. 1b). In addition, the evaluation of the lung coefficients and Ashcroft scores of pulmonary fibrosis showed that Rg3 relieved lung tissue damage caused by lung fibrosis (Fig. 1c, d). Immunohistochemical was performed to analyse SMA and HIF-1α expression. The results showed that the expression of SMA was decreased after Rg3 treatment, and the inhibitory effect of Rg3 on HIF-1α was weaker than that of SMA (Fig. 1e). After analyzing the results of IHC staining, it was found that Rg3 had a greater inhibitory effect on the expression of HIF-1α in the nucleus (Fig. 1f). This finding indicates that the role of Rg3 in pulmonary fibrosis is likely related to hypoxia.

### Ginsenoside Rg3 inhibits the bleomycin-promoted migration of fibroblasts by preventing HIF-1α nuclear localisation

**Fig. 2:**
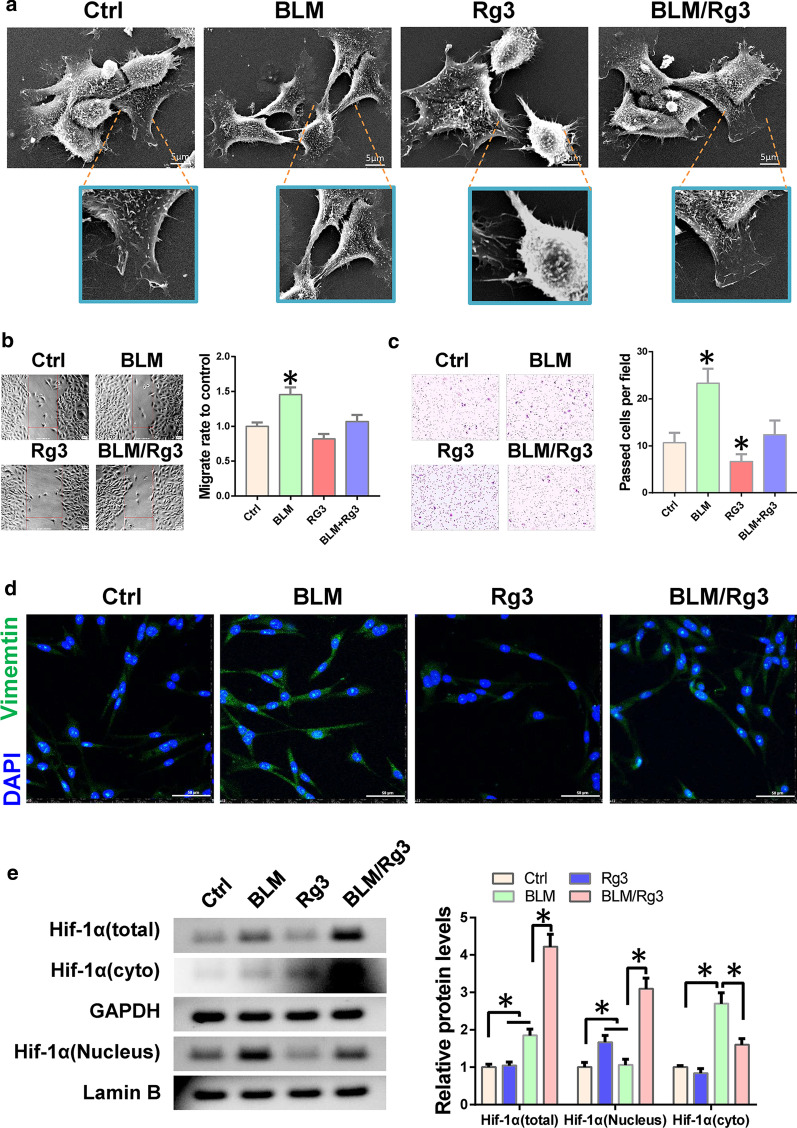
Rg3 inhibits fibroblast migration and invasion. Fibroblast cell LL 29 were treated with bleomycin alone or simultaneously with Rg3. **a** Cell phenotype was detected by SEM. **b** The effect of Rg3 on cell migration ability was detected using wound healing. **c** The effect of Rg3 on cell invasion was detected by transwell assay. **d** Immunofluorescence is used to detect Vimentin expression. **e** The expression of HIF-1α in the nucleus and cytoplasm was detected by western blot

To verify how Rg3 inhibits pulmonary fibrosis, we treated LL 29 lung fibroblasts with bleomycin. Results of scanning electron microscopy indicated that cells became spindle-shaped and their adhesion decreased when treated with bleomycin. While, the EMT phenotype was suppressed after treatment with Rg3 (Fig. 2a). Next, we performed transwell and wound healing assays to detect cell invasion and migration ability. The results showed that bleomycin enhanced the invasion and migration ability of LL 29 cells, whereas Rg3 inhibited this effect (Fig. 2b, c). Immunofluorescence results also showed that Rg3 can inhibit the expression of Vimentin (Fig. 2d). Given that the role of HIF-1α in EMT is mainly achieved through transcriptional regulation, we examined the expression of HIF-1α in the nucleus and cytoplasm. Western blot results showed that bleomycin induced the expression of HIF-1α and promoted its nuclear localisation, whereas Rg3 inhibited the nuclear localisation of HIF-1Α. We speculate that Rg3 may inhibit the migration and invasion of fibroblasts by inhibiting the nuclear localisation of HIF-1α (Fig. 2e).

### Ginsenoside Rg3 binds HIF-1α to inhibit EMT evolution in fibroblasts

**Fig. 3:**
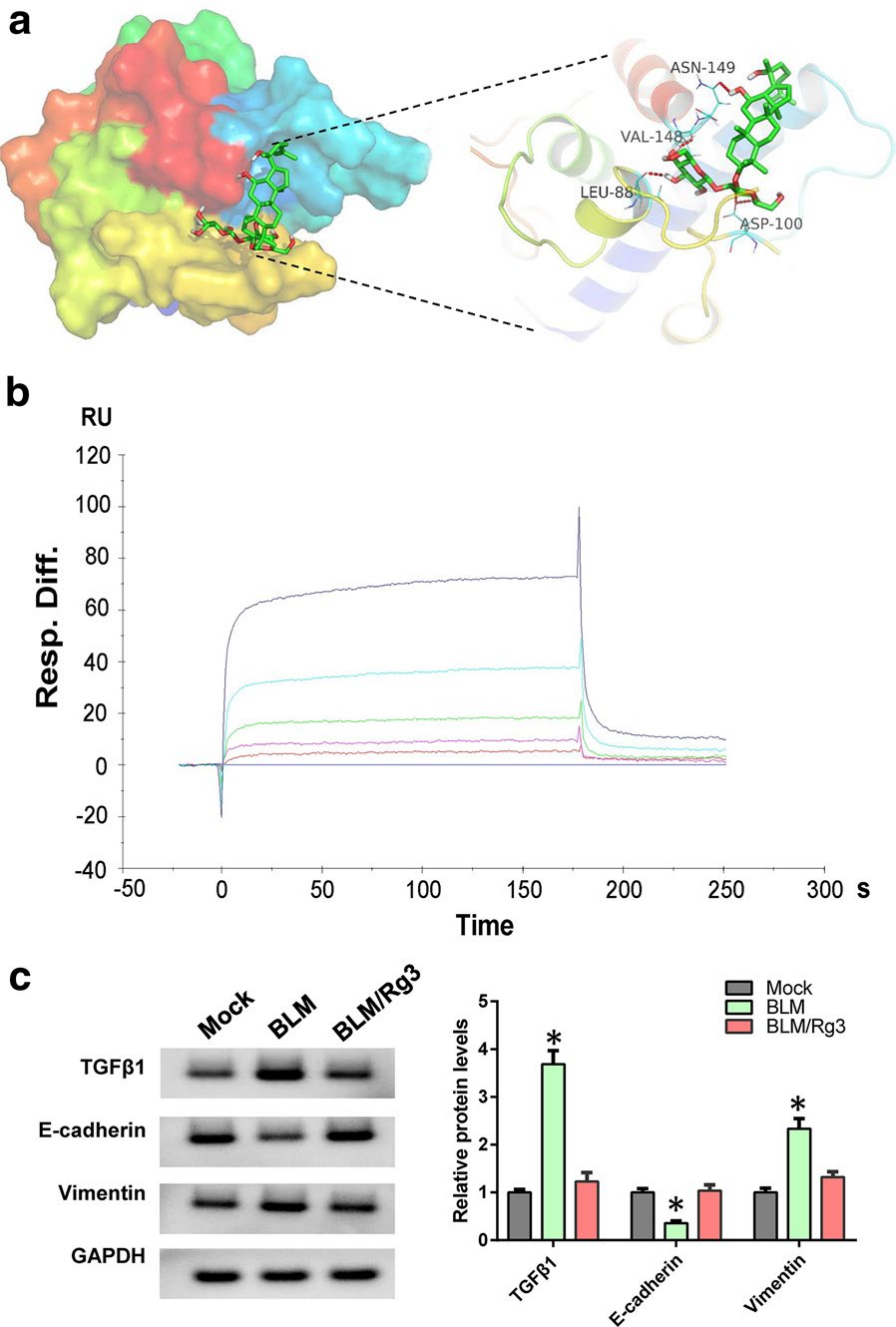
Rg3 can directly bind HIF-1α. **a** Molecular docking between Rg3 and HIF-1α. **b** Biacore was used to analyse the binding ability of Rg3 to HIF-1α. **c** Western blot was used to detect the expression levels of TGFβ1 and EMT markers

To investigate if Rg3 directly binds HIF-1α and thus inhibits its role in fibroblasts, we performed molecular docking to simulate the interaction and found a strong binding force between Rg3 and HIF-1α (Fig. 3a). Subsequent Biacore experiments further verified the binding of Rg3 to HIF-1α (Fig. 3b). These results show that Rg3 can bind to HIF-1α. Whether Rg3 binds to HIF-1α and prevents the nuclear localization of HIF-1α, we use western blot to detect. Results showed that Rg3 inhibited the expression of TGFΒ1 and Vimentin but increased the expression of E-cadherin (Fig. 3c).

### Up-regulation of HIF-1α can reverse the inhibitory effect of ginsenoside Rg3 on fibroblasts

**Fig. 4:**
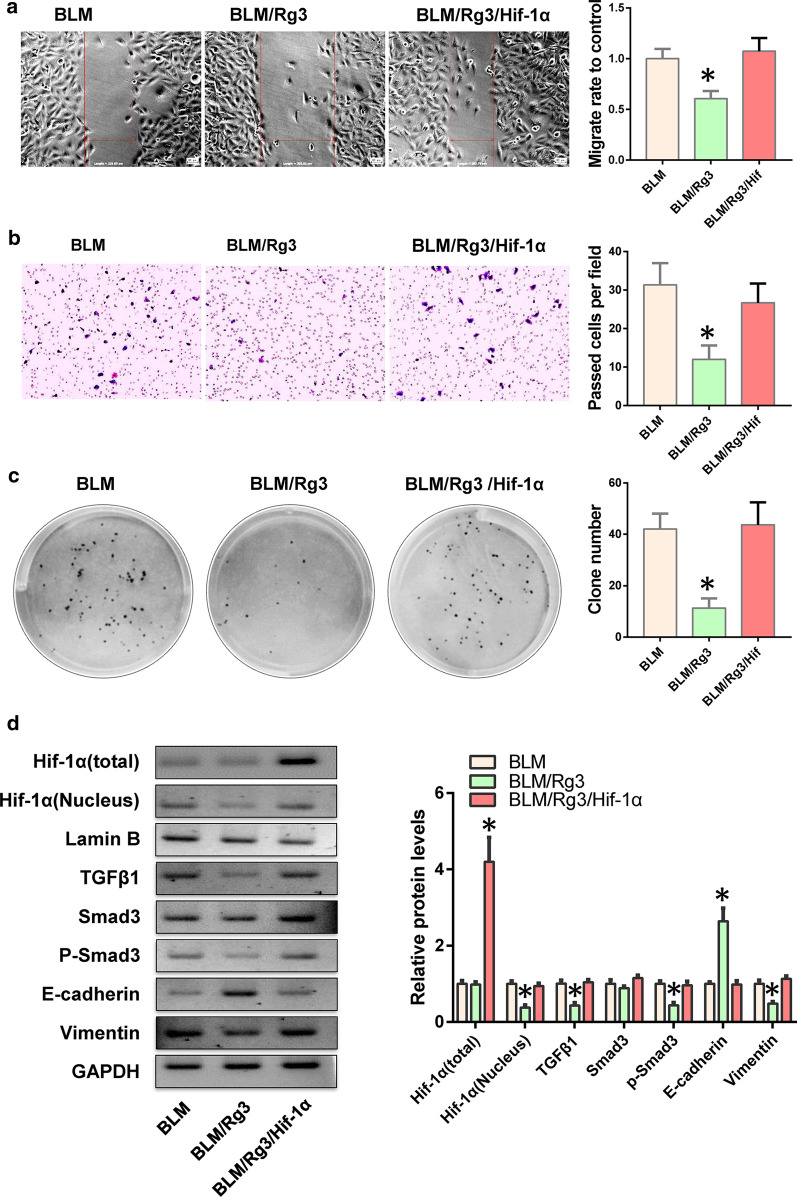
HIF-1α promotes the EMT process of fibroblasts. HIF-1α was overexpressed in Rg3-treated fibroblasts, and EMT-related functions were detected. **a** Cell migration ability was detected via wound healing assay. **b** Transwell assay was used to determine cell invasion. **c** Cell proliferation were tested through clone formation experiments. **d** Western blot was utilised to detect the expression of the TGFΒ1/Smad3 pathway and EMT markers

To confirm that Rg3 inhibits the EMT evolution of fibroblasts through HIF-1α, we overexpressed HIF-1α in Rg3-treated fibroblasts. We found that HIF-1α overexpression reversed the inhibitory effect of Rg3 on fibroblast migration and invasion (Fig. 4a, b). We also performed clone formation experiments and found that HIF-1α restored the fibroblasts’ proliferative ability (Fig. 4c). Results of western blot indicated that HIF-1α could resist the inhibitory effect of Rg3 on its nuclear localisation, thereby promoting the process of EMT through the TGFB1/Smad3 pathway (Fig. 4d).

### HIF-1α counteracts the effects of ginsenoside Rg3 and accelerates bleomycin-induced pulmonary fibrosis

**Fig. 5:**
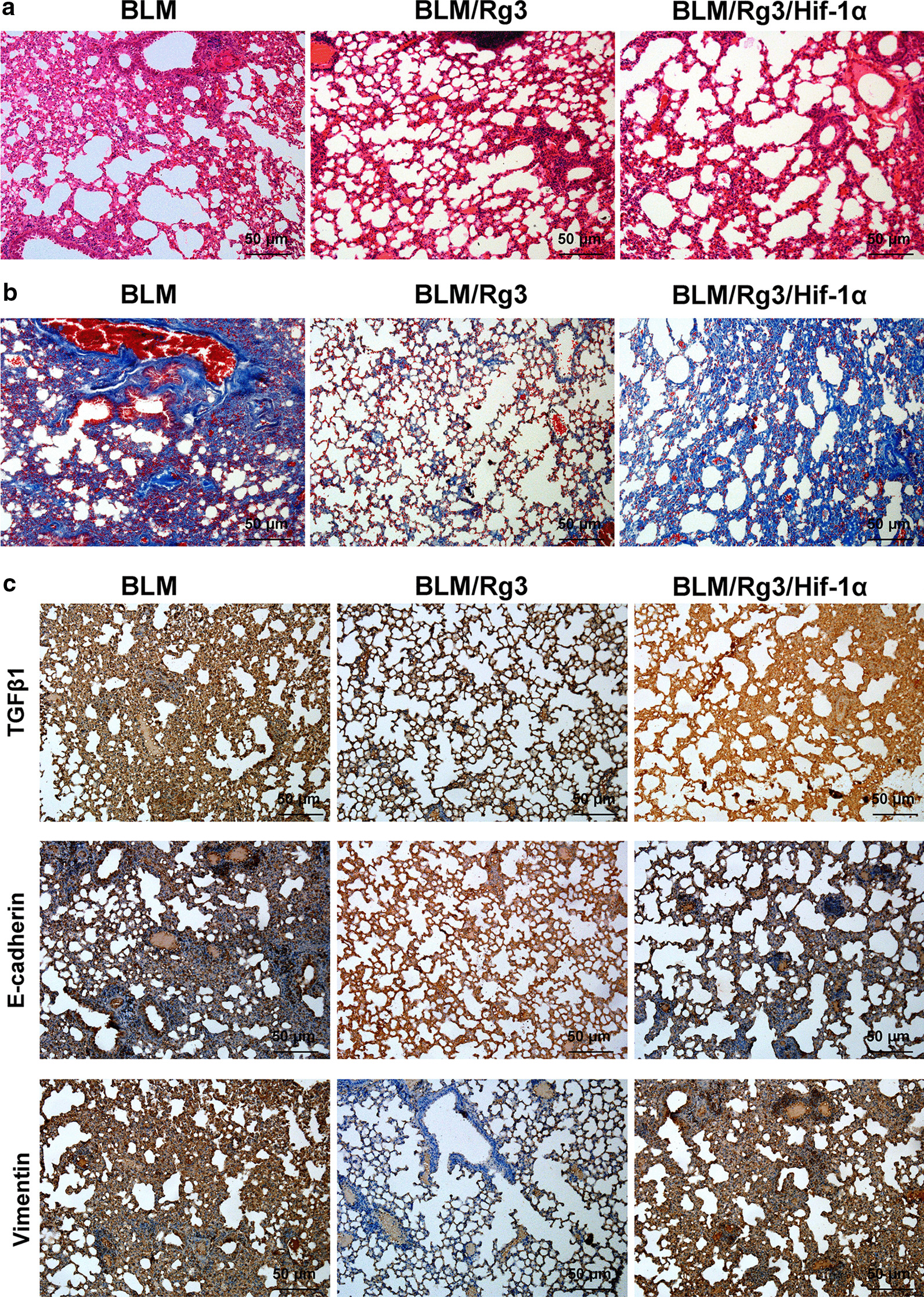
HIF-1α reverses the inhibitory effect of Rg3 on fibrosis. We overexpress HIF-1α in mice with bleomycin-induced pulmonary fibrosis after treatment with Rg3, and then evaluate the degree of pulmonary fibrosis. **a** HE staining was performed to detect the morphology of lung tissue (× 100 magnification). **b** Masson staining was conducted to detect the proportion of fibroblasts (× 100 magnification). **c** Immunohistochemistry was applied to observe the expression of TGFΒ1 and EMT markers (× 100 magnification)

The above experiments showed that Rg3 inhibited pulmonary fibrosis by inhibiting the HIF-1α/TGFΒ1 signalling pathway. Here, we randomly divided 18 mice into three groups and used bleomycin to induce pulmonary fibrosis model. Then we overexpressed HIF-1α by AAV in Rg3-treated lung fibrosis animals. HE and Masson staining revealed that the expression of HIF-1α accelerated the lung fibrosis inhibited by Rg3 (Fig. 5a, b). The immunohistochemical staining results showed that HIF-1α promoted the expression of TGFΒ1 and Vimentin and inhibited the expression of E-cadherin (Fig. 5c). All experiments showed that the inhibitory effect of Rg3 on bleomycin-induced pulmonary fibrosis was achieved by preventing the nuclear localisation of HIF-1α, thus inhibiting the TGFΒ1-mediated EMT process.

## Discussion

The pathophysiological process of many diseases is accompanied by hypoxia. The hypoxic microenvironment is a double-edged sword. May cause stress response and damage human organs [18]. During tumour and lung fibrosis, hypoxia stimulation leads to the excessive proliferation of tumour cells and fibroblasts, which accelerates the progression and deterioration of the disease [19]. HIF-1α plays an important role in the occurrence and development of many diseases as a response factor to hypoxia. In tumours, HIF-1α can trigger the transcription of a series of oncogenes and promote the malignant progression of tumours [20, 21]. HIF-1α is also widely involved in fibrosis in various tissues. In the kidney, the HIF-1α signalling pathway is believed to play a role in the early onset of renal fibrosis [22]. HIF-1α can also participate in liver fibrosis by regulating liver sinusoidal endothelial cells [23]. In this study, we found that bleomycin induction can increase HIF-1α expression and nuclear localization. Subsequently, HIF-1α activated TGFβ1/Smad3 signalling pathway to activate EMT in fibroblasts, which ultimately leads to enhanced cell proliferation and migration capacity and excessive fibroblast proliferation. We used ginsenoside Rg3 treatment and found that it can prevent the nuclear localisation of HIF-1α and inhibit the EMT-mediated cell proliferation. Results of molecular docking and Biacore experiments suggested that ginsenoside Rg3 can bind HIF-1α. This provides molecular evidence that Rg3 may prevent the nuclear localization of HIF-1α. Although we found that Rg3 can treat pulmonary fibrosis induced by bleomycin, it has no obvious effect in animals with pulmonary fibrosis induced by paraquat. This seems to indicate that Rg3 seems to be only suitable for early intervention of pulmonary fibrosis. If the condition is more serious or the course of the disease progresses too quickly, the efficacy of Rg3 may be less effective (Additional file 1).

EMT is a process in which epithelial cells lose their polarity, reduce their adhesion ability, and acquire mesenchymal cell characteristics. EMT is widely involved in a series of physiological processes, including embryonic development, wound healing and tumour progression [24–26]. Extensive research evidence has indicated that EMT is crucial in fibrosis remodelling [27, 28]. In radiation-induced pulmonary fibrosis, the interaction of Foxm1 and Snail1 activates TGFβ1-induced EMT and promotes pulmonary fibrosis [29]. Similarly, in vitro and in vivo blocking of TGF-β can reduce the progression of bleomycin-induced pulmonary fibrosis [30].

TGFβ1/Smad is a key signal element that regulates EMT. It affects cell proliferation, differentiation, apoptosis and extracellular matrix production to regulate tissue morphogenesis and differentiation [31]. TGFβ1 can increase the expression of the mesenchymal cell marker Vimentin and decrease the expression of the epithelial cell marker E-cad [32, 33]. In this study, we found that bleomycin induced an increase in the nuclear localisation of HIF-1α and activated the TGFβ1/Smad signalling pathway, which in turn led to the excessive proliferation of fibroblasts mediated by EMT. After ginsenoside Rg3 treatment, the nuclear localization of HIF-1α decreases, thereby slowing down the process of pulmonary fibrosis.

## Conclusion

Overall, ginsenoside Rg3 can inhibit BLM-induced pulmonary fibrosis. The mechanism is attributed to the ability of ginsenoside Rg3 to reduce the nuclear localisation of HIF-1α and inhibit the evolution of EMT-related pulmonary fibrosis mediated by the TGF-β1/Smad3 signalling pathway. This research is expected to provide experimental evidence for the use of clinical hypoxic treatment methods, such as ginsenoside Rg3, in pulmonary fibrosis.

## Supplementary Information

**Additional file 1.** Original gel images of western blot.

## Data Availability

The datasets used during the current study are available from the corresponding author on reasonable request.

## Abbreviations

HIF-1α: Hypoxia inducible factor alpha
EMT: Epithelial mesenchymal transition
IHC: Immunochemistry
