# Supplementary material for: Ginsenoside Rg3 inhibits pulmonary fibrosis by preventing HIF-1α nuclear localisation
Source: BMC Pulm Med. 2021 Feb 27;21:70. doi: 10.1186/s12890-021-01426-5 (PMC7912494; doi:10.1186/s12890-021-01426-5)
Supplement: Supplementary file 1 — Additional file 1. Original gel images of western blot. [file 12890_2021_1426_MOESM1_ESM.pdf]

Fig.2

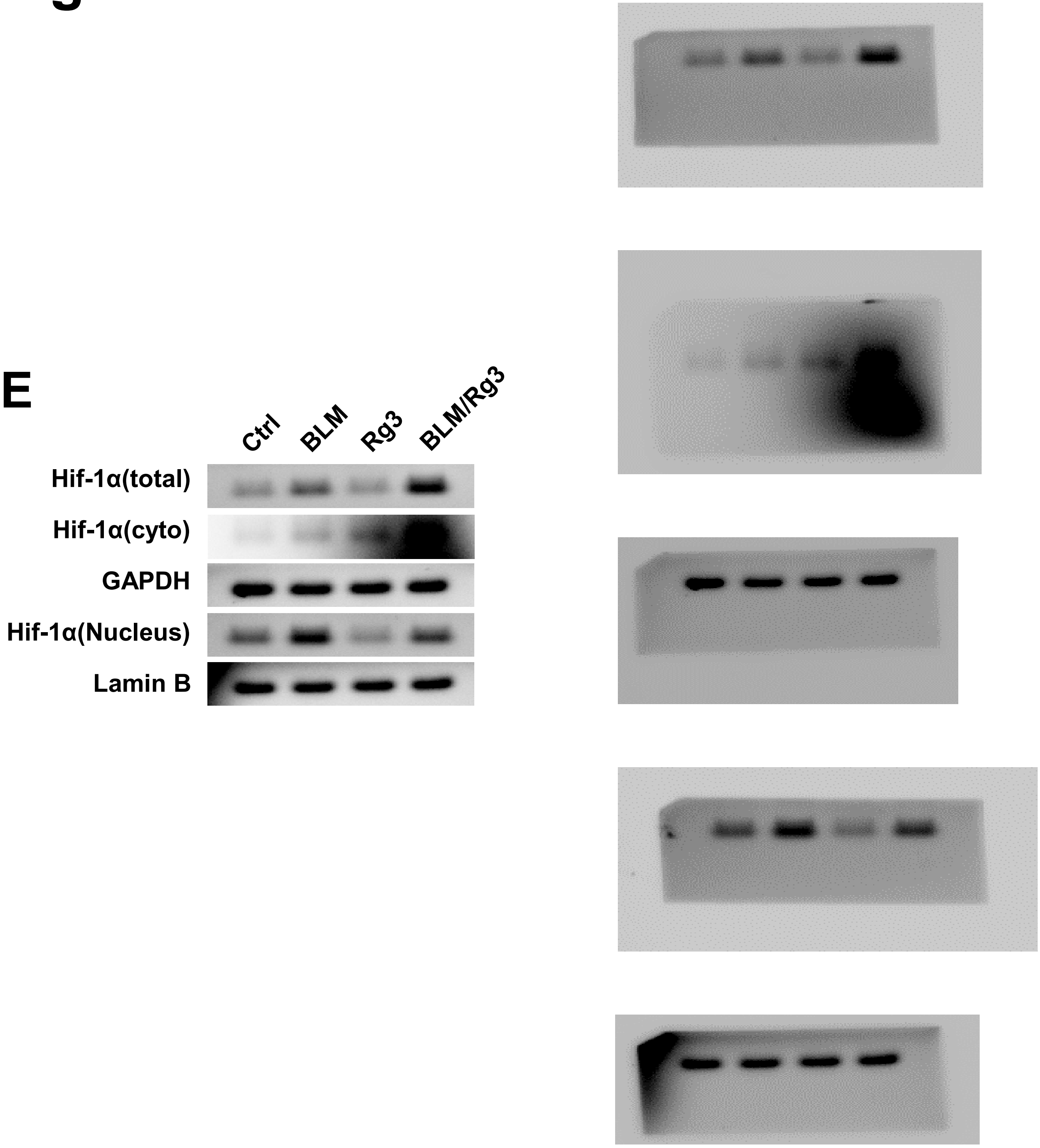

Fig. 2 Rg3 inhibits fibroblast migration and invasion. Fibroblast cell LL 29 were treated with bleomycin alone or simultaneously with Rg3. (A) Cell phenotype was detected by SEM. (B) The effect of Rg3 on cell migration ability was detected using wound healing. (C) The effect of Rg3 on cell invasion was detected by transwell assay. (D) Immunofluorescence is used to detect Vimentin expression. (E) The expression of HIF-1α in the nucleus and cytoplasm was detected by western blot.

Fig.3

C

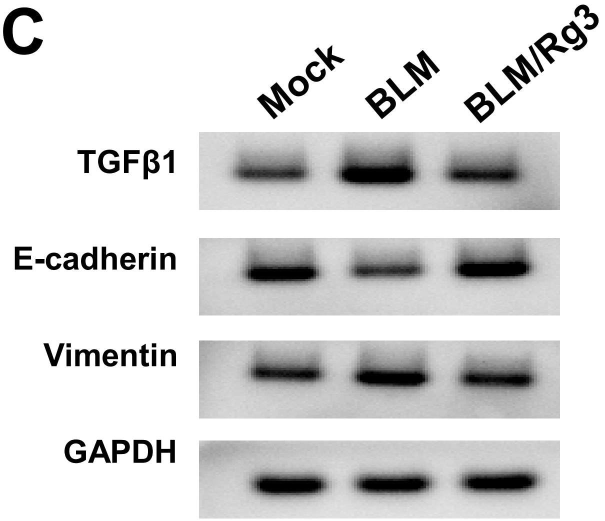

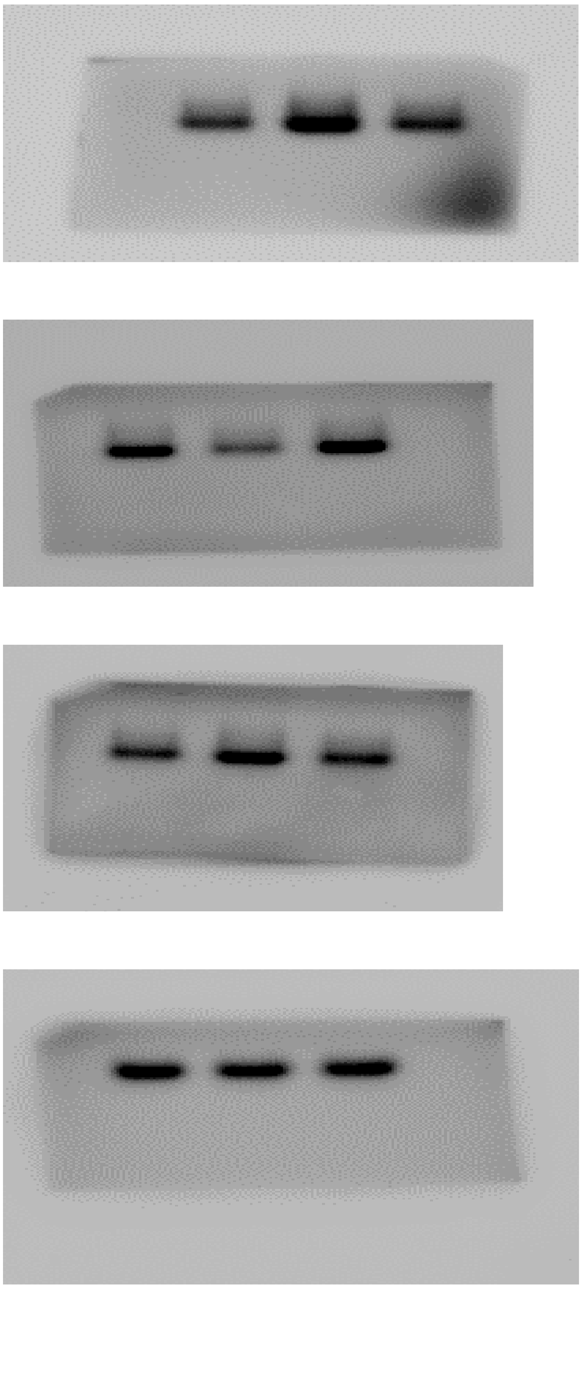

Fig. 3 Rg3 can directly bind HIF-1α. (A) Molecular docking between Rg3 and HIF-1α. (B) Biacore was used to analyse the binding ability of Rg3 to HIF-1α. (C) Western blot was used to detect the expression levels of TGFβ1 and EMT markers.

Fig.4

D

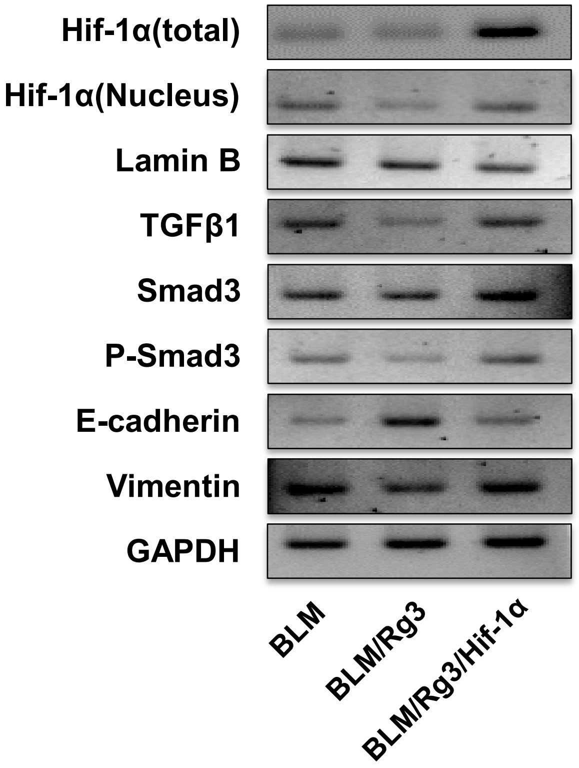

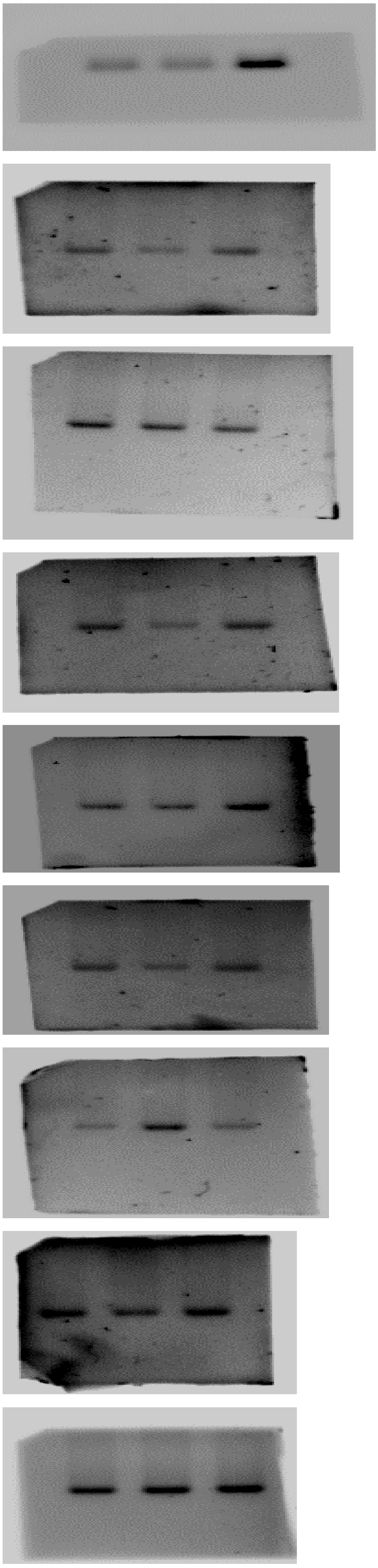

Fig. 4 HIF-1α promotes the EMT process of fibroblasts. HIF-1α was overexpressed in Rg3-treated fibroblasts, and EMT-related functions were detected. (A) Cell migration ability was detected via wound healing assay. (B) Transwell assay was used to determine cell invasion. (C) Cell proliferation were tested through clone formation experiments. (D) Western blot was utilised to detect the expression of the TGFB1/Smad3 pathway and EMT markers.
